# Renal Angiomyolipoma with Fatty Thrombus Extending to the Right Atrium: An Exceptional Presentation

**DOI:** 10.1155/2013/120383

**Published:** 2013-04-16

**Authors:** Yassine Nouira, Yousri Kallel, Mourad Gargouri, Ahmed Sellami, Rami Boulma, Jalel Ziedi, Mohamed Chelif, Sami Ben Rhouma, Taoufik Kalfat, Adel Khayati

**Affiliations:** ^1^Department of Urology, La Rabta University Hospital, 1007 Tunis, Tunisia; ^2^Department of Cardiac Surgery, La Rabta University Hospital, 1007 Tunis, Tunisia

## Abstract

This paper reports the case of 34-year-old woman who presented with bilateral renal angiomyolipomas (AMLs). On the right side, there was a large AML with a fatty thrombus extending to the right atrium. The treatment consisted of right nephrectomy and complete thrombectomy with extracorporeal circulation and right atriotomy. Postoperatively, the patient was septic and died on postoperative day 7 because of septic shock.

## 1. Introduction

Renal angiomyolipomas (AMLs) are benign renal tumors that warrant only excision when symptomatic or large. The major known complication of these tumors is rupture and retroperitoneal bleeding that may be massive and threatens the patient's life [1].

Herein, we report a case depicting the dangerousness of these tumors with a fatty thrombus of the inferior vena cava extending to the right atrium.

This is a rare and serious presentation of renal AML.

## 2. A Case Report

**Figure 1:**
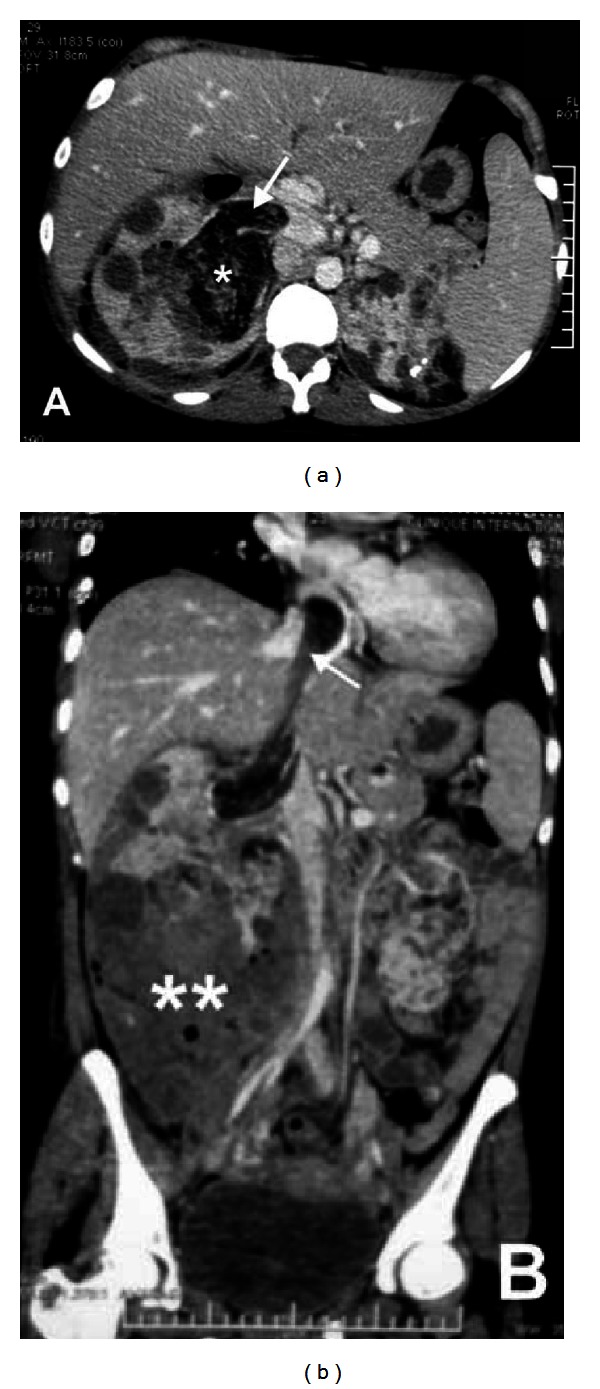
(a) Coronal CT scan showing renal AML (∗) with inferior vena cava thrombus (arrow). (b) The thrombus extends up to right atrium. Note the presence of a large perirenal infected hematoma due to an old bleeding from lower pole AML (∗∗).

A 34-year-old woman, with unremarkable medical history, presented with right lumbar pain beginning two weeks previously. Physical examination revealed a 20 cm tender right lumbar mass with no fever. No skin lesions were noted, and blood pressure was 120/70 mmHg. CT scan showed multiple bilateral AML with various sizes with one 8 cm perihilar lesion with a fatty thrombus extending into the inferior vena cava to the right atrium. Also, there was evidence of old hematoma in the renal fossa extending in the pelvis (Figure 1).

Given this unusual extension of the tumor and the risk that a part of the thrombus may detach and cause pulmonary embolism, rapid surgical intervention was decided.

The patient was first operated through a chevron incision, the right colon was reflected, and the whole right kidney was dissected. The right renal artery was ligated, and the kidney was left attached only by the renal vein with the thrombus inside. At the level of the lower pole of the kidney, there was a 15 cm infected hematoma that was evacuated and cleaned.

A sternotomy was then performed, and extracorporeal circulation was installed; the right atrium was opened, and the distal part of the thrombus was visualized.

**Figure 2:**
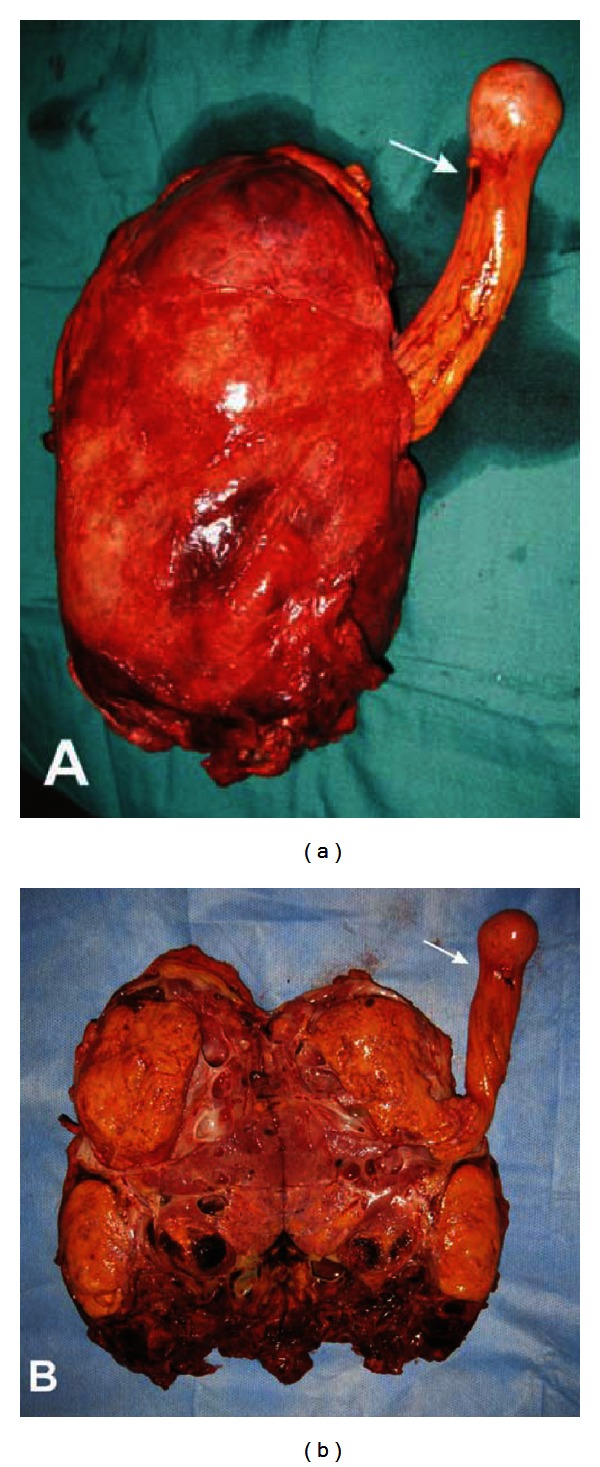
(a) Operative specimen showing the renal AML with the fatty thrombus extended from it. (b) Opened specimen showing the fatty thrombus in continuity with the AML. Note the notch in the distal part of the thrombus corresponding to the intracardiac passage (arrow).

**Figure 3:**
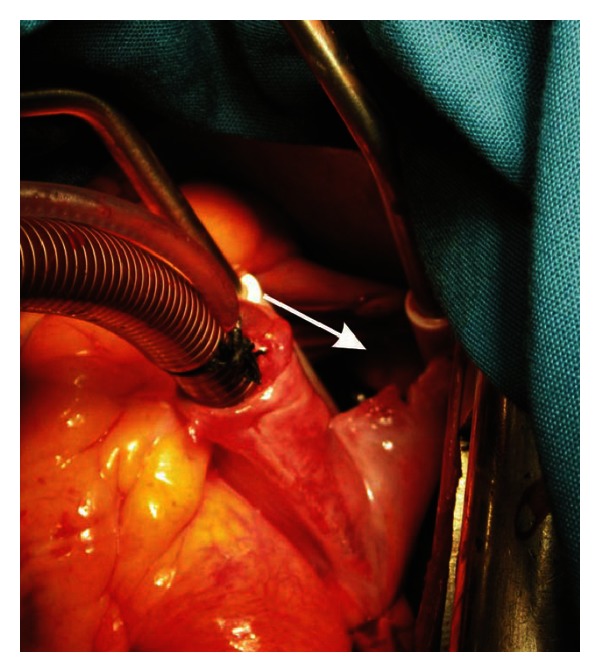
Opened right atrium showing the intra-atrial part of the thrombus (arrow).

A circumferential incision of the renal vein was done, and the kidney was extracted along with the fatty thrombus attached (Figure 2) under visual control from the right atrium (Figure 3).

Immediate postoperative course was uneventful; however, the patient remained septic and kept under ventilation assistance. The patient died on postoperative day 7 by septic shock.

## 3. Discussion

The case we report is the perfect demonstration that although renal AMLs are benign, they can be very serious lesions. Inferior vena cava involvement is present in 4% to 10% of renal cancer [2], and this extension is rarely reported in AML. Only 47 cases of renal AML with endovascular lesions have been reported so far [3], and, to the best of our knowledge, this is the fifth case of right atrial involvement [4–6].

Surgical treatment of renal AML is usually conservative and is limited to tumor excision and elective hemostasis of its nourishing vessels. Complete tumor excision is the warranty of the lack of tumor recurrence. In case of endovascular extension, nephrectomy and thrombectomy are the treatment of choice.

 Thrombus extension into the heart is considered a high surgical difficulty. Opening of the right atrium to control the distal part of the fatty thrombus is necessary as the intracardiac part is fragile at the level of its passage through the ostium of vena cava as demonstrated in the case we report by the presence of a distal notch delineating the intracardiac part of the thrombus (Figure 2).

Although surgical procedure was very satisfying in our case, the patient had fatal issue, and this can be explained by the presence of an infected perirenal hematoma with intraoperative bacterial discharge favored by the extracorporeal circulation and postoperative sepsis. 

## 4. Conclusions

Renal AML is exceptionally associated with endovascular extension. Fat thrombus may extend from the tumor to the inferior vena cava up to the right atrium. The present case shows that this benign lesion can be very serious by its endovascular extension and can be potentially lethal to the patient.
